# Historical account on gaining insights on the mechanism of crown gall tumorigenesis induced by *Agrobacterium tumefaciens*

**DOI:** 10.3389/fmicb.2014.00340

**Published:** 2014-08-07

**Authors:** Clarence I. Kado

**Affiliations:** Davis Crown Gall Group, Department of Plant Pathology, University of California, DavisDavis, CA, USA

**Keywords:** Ti plasmid, *Agrobacterium*, T pilus, T-DNA, type IV secretion system, type VI secretion system, opines, conjugative transfer

## Abstract

The plant tumor disease known as crown gall was not called by that name until more recent times. Galls on plants were described by Malpighi (1679) who believed that these extraordinary growth are spontaneously produced. *Agrobacterium* was first isolated from tumors in 1897 by Fridiano Cavara in Napoli, Italy. After this bacterium was recognized to be the cause of crown gall disease, questions were raised on the mechanism by which it caused tumors on a variety of plants. Numerous very detailed studies led to the identification of *Agrobacterium tumefaciens* as the causal bacterium that cleverly transferred a genetic principle to plant host cells and integrated it into their chromosomes. Such studies have led to a variety of sophisticated mechanisms used by this organism to aid in its survival against competing microorganisms. Knowledge gained from these fundamental discoveries has opened many avenues for researchers to examine their primary organisms of study for similar mechanisms of pathogenesis in both plants and animals. These discoveries also advanced the genetic engineering of domesticated plants for improved food and fiber.

## Introduction

**Figure 1:**
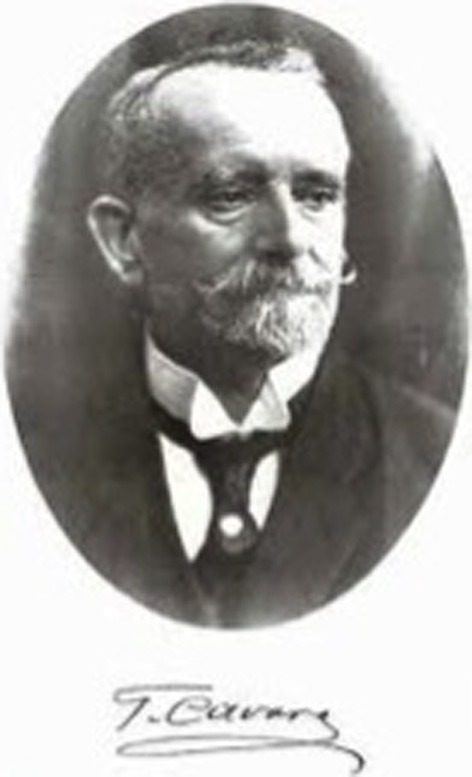
**Fridiano Cavara**.

**Figure 2:**
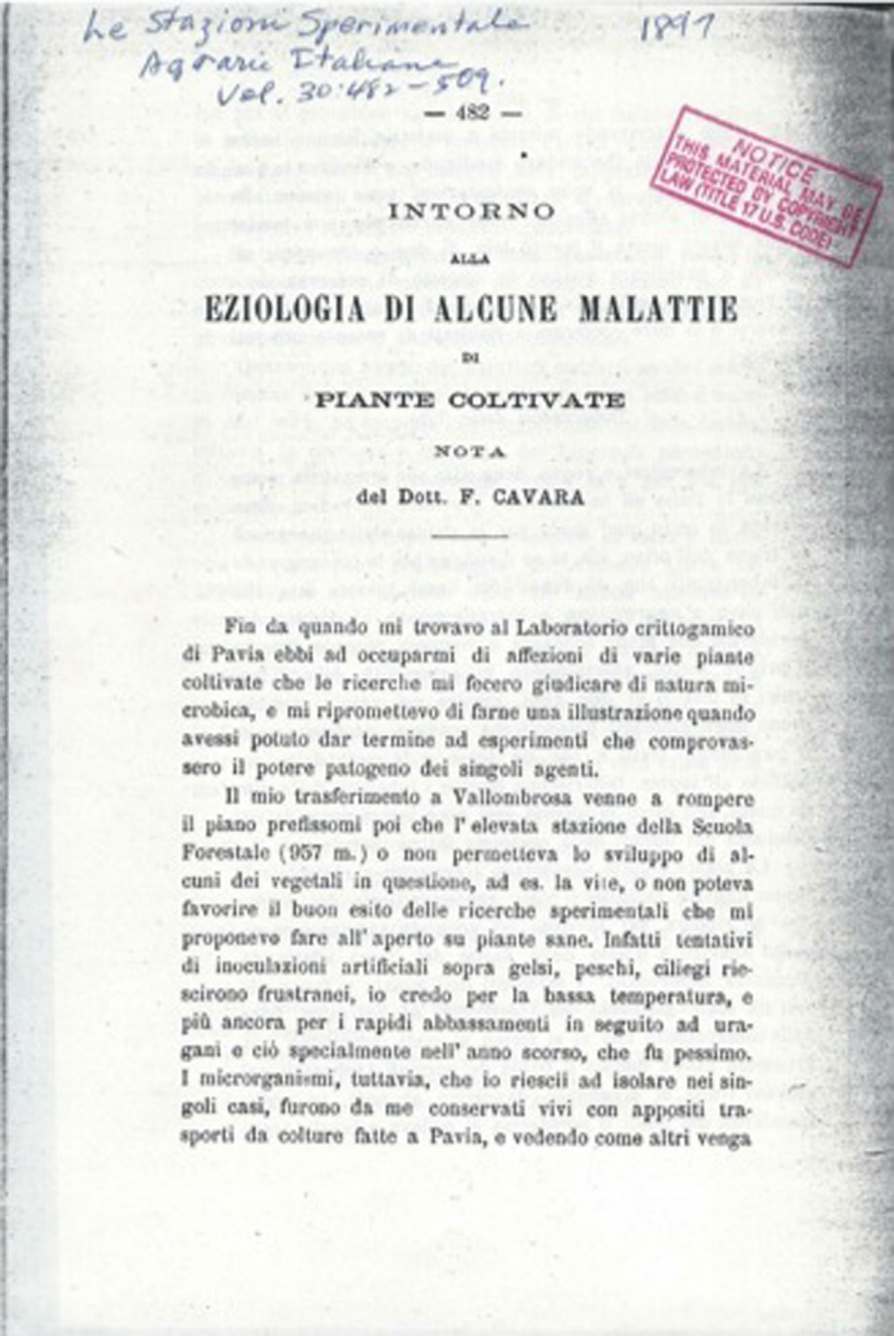
**Paper by Fridiano Cavara in 1897 describing galls on grapevines from which he isolated the tumorigenic bacterium and demonstrated its gall forming activity on young grapevines**.

**Figure 3:**
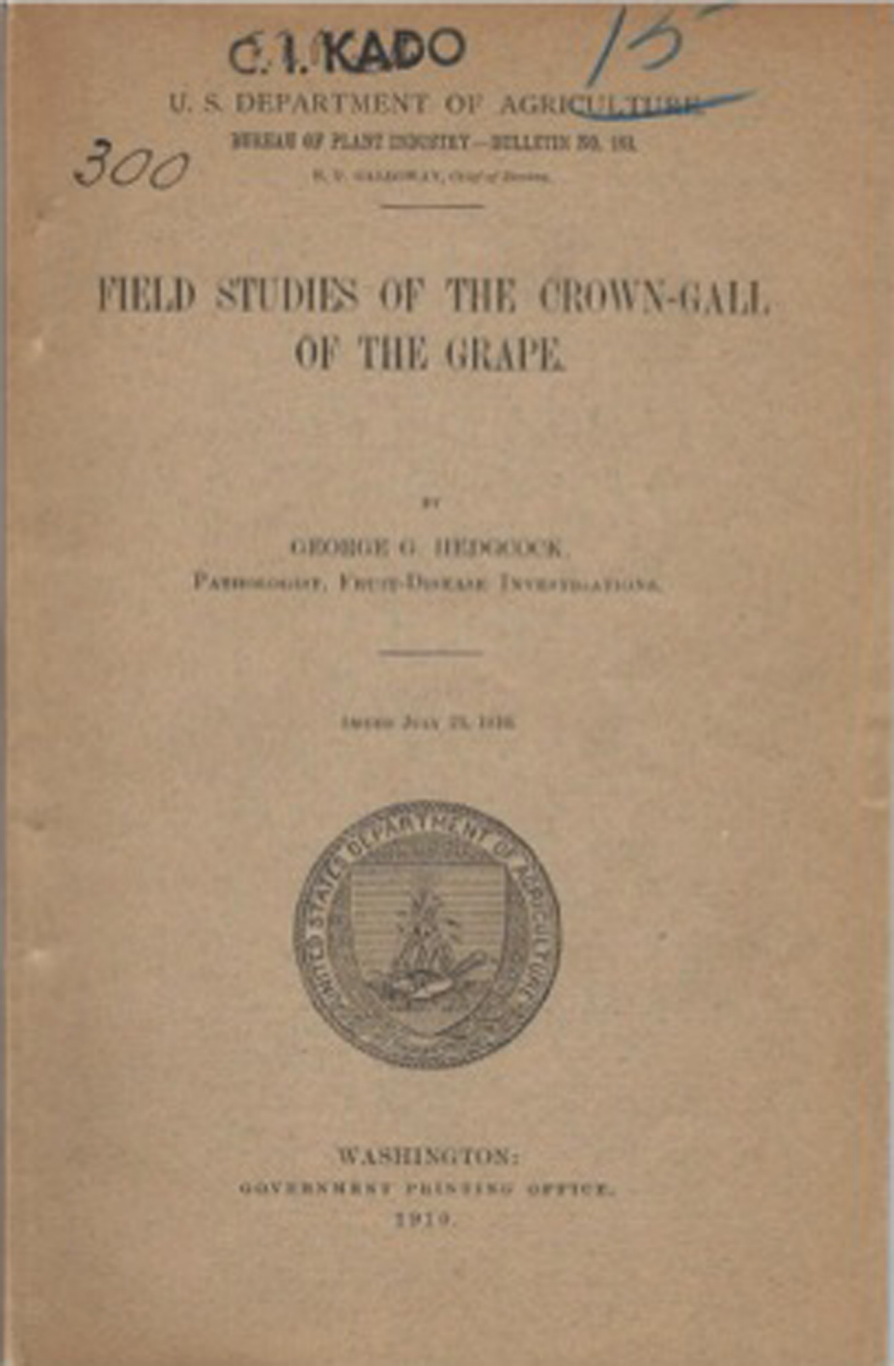
**A compendium by George Hedgcock on crown gall of grapevines published in 1910 describing his 1904 work on the isolation of crown gall producing bacterium and demonstrating tumorigenicity**.

Crown gall is a name given to abnormal tumor-like growths often observed at the base of the trunk and roots of trees, grapevines, and woody plants. The nature of the cause of crown gall was unknown before 1897. Not referenced by many authors who worked on this disease was the published work of Fridiano Cavara (Figure 1). He described in detail the galls formed at the base of grapevines that were in the Royal Botanical Gardens of Napoli (Naples), Italy. More importantly, he also described the isolation of a bacterium that he showed caused similar tumors on young grapevines. This work was published in Le Stazioni Sperimentale, Agrari Italiane (Cavara, 1897a,b; Figure 2). In 1904, George C. Hedgcock reported the isolation of a causal bacterium from grapevine galls that he described in a US Department of Agriculture Bureau of Plant Industry bulletin (Hedgcock, 1910, p. 21; Figure 3). His monograph remains not frequently cited. Most cited as allegedly the first to isolate the causal bacterium was Smith and Townsend (1907). The authors named the causal organism *Bacterium tumefaciens*. E. F. Smith had visited Cavara in Naples and learned how to isolate the causal bacterium from grapevine galls (Rodgers, 1952). He and C. O. Townsend then published the isolation of the crown gall causing bacterium from chrysanthemum. Smith worked extensively on the disease and showed that *B. tumefaciens* can induce gall formation in a number of herbaceous plants (Smith, 1911b). Subsequently, the name *B. tumefaciens* was changed briefly to *Pseudomonas tumefaciens* (Duggar, 1909) and then to *Phytomonas tumefaciens* (Bergey et al., 1923), followed by *Polymonas tumefaciens* (Lieske, 1928), and to *Agrobacterium tumefaciens* (Conn, 1942). The varying phases of the life cycle of *P. tumefaciens* were described by Stapp and Bortels (1931).

In France, Fabre and Dunal (1853) named the tumors observed on diseased grapevines as “broussin.” Dornfield (1859) called the galls found on grapevines in Germany as “Grind,” but the gall disease was also called “Ausschlag,” “Mauche,” “Krebs,” “Kropf,” “Raude,” and “Schorf.” In Italy, the gall disease on grapevines was called “rogna” (Garovaglio and Cattaneo, 1879) and “tubercoli” (Cavara, 1897a,b). In the United States, the gall disease observed on grapevines was called “black-knot” (Galloway, 1889) and likewise in Canada (Fletcher, 1890). Other names such as tubercular galls were applied to this tumorous disease that had become recognized throughout the continents wherever grapevines and woody crops were cultivated.

Eventually, nurserymen, farmers, viticulturalists, etc., became aware of the gall producing disease that occurred at the base of trees and vines near the junction of the roots to the trunk, known to these growers as the “crown,” the term “crown-gall” became the common name used to recognize the tumor-forming disease.

## Search for the agent that caused crown gall

Once *A. tumefaciens* was established as the cause of crown gall, the quest was initiated for the mechanism by which this pathogen induced tumors in plants. It was widely known that *A. tumefaciens* induces tumors readily by mechanical inoculation of many different plant species. Eventually, over 90 families of plants were found to be susceptible to Crown Gall disease incited by this bacterium (Kado, 2010). In Nature, however, crown gall is found mainly on woody plants such as stone fruit trees of the genus *Prunus* and other members of the Rosaceae (rose) family, members of the Vitaceae (grape), and members of the Juglandaceae (walnut) family. There are at least 41 families of plants found to be naturally infected by *A. tumefaciens* (Kado, 2010). Experimental inoculations with *A. tumefaciens* on susceptible herbaceous plants have provided excellent opportunities to study in detail the timing of cellular transformation and the process of tumor formation.

Three schools of thought on the cause of crown gall were proposed. (1) *A. tumefaciens* caused tumors by producing one or more irritating chemicals that promoted tumor formation. (2) The phytohormone auxin was believed to play a central role in tumor formation and development. (3) Plant hosts were conditioned by *A. tumefaciens* to initiate and promote tumor formation by a tumor-inducing principle (Braun and Mandle, 1948).

### *A. tumefaciens* produces chemical irritants that led to tumor formation in plant hosts

**Figure 4:**
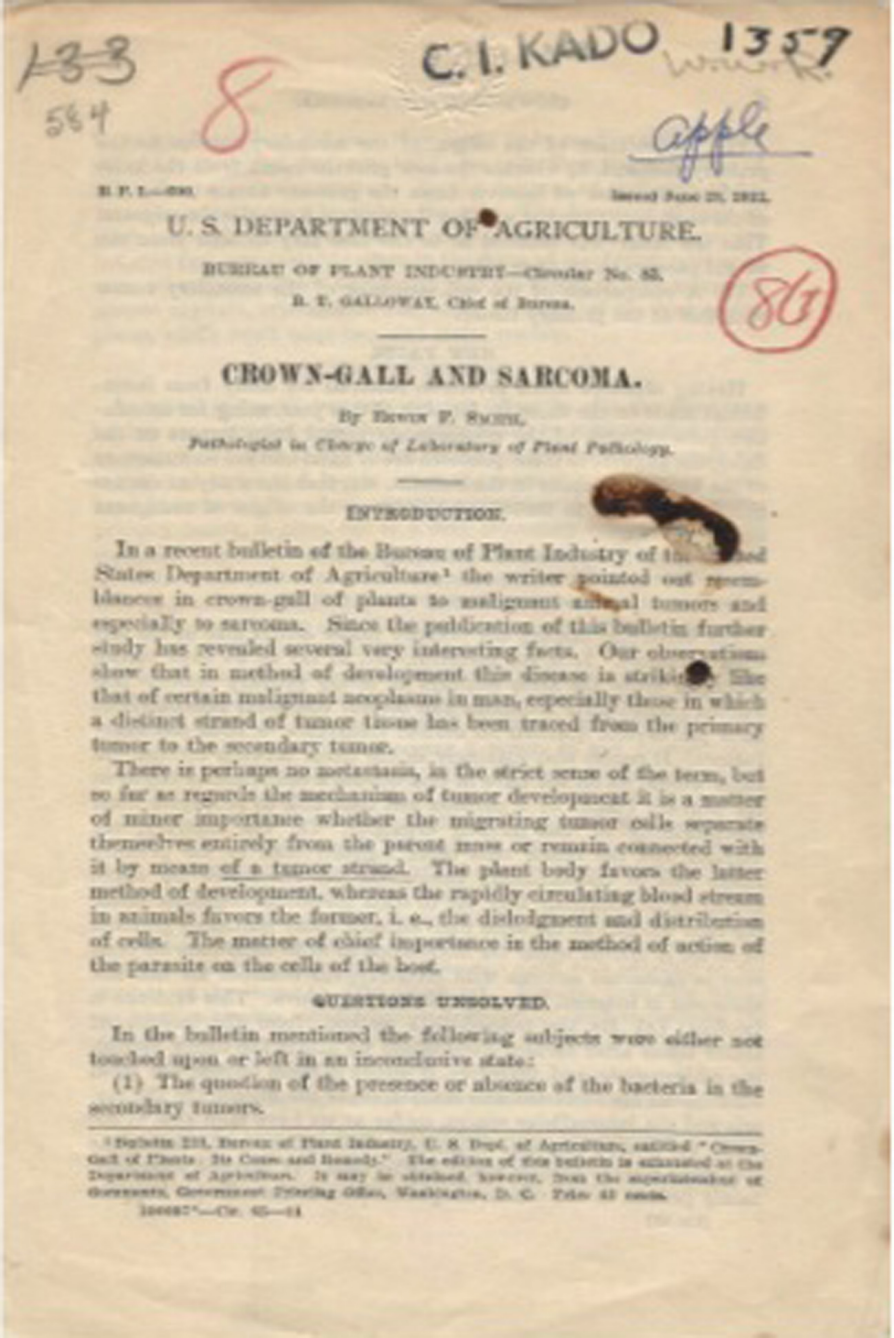
**Paper published in 1911 by Erwin F. Smith describing the similarities and differences between crown gall and human sarcoma**.

Normally, plant cells grow, develop, and multiply under stringent control. There is a mutual balance and restraint to maintain cellular order and differentiation. On the other hand, crown gall cells multiply and give rise to tissues that are not self-limiting and tax the surrounding cellular community of their energy and resources. So, the question arose among many researchers of that era, what is it that gives crown gall cells these perverse properties? In the medical field, at that early period of cancer research, it was believed that cancer was caused by some forms of external irritants. In fact, analogies between human sarcoma and crown gall were put forth by Smith (1911a; Figure 4).

In 1917, Smith used castor bean (*Ricinus communis* L.), a member of the Euphorbiaceae (spurge family), as the host for *A. tumefaciens* (called at that time *B. tumefaciens* Sm. and T.) to determine the mechanism of crown gall tumor growth (Smith, 1917). After a large number of tests both physical and chemical, Smith hypothesized that “dilute ammonia causes intumescences and have rendered it probable that ammonia liberated within the cell in small quantities by the imprisoned bacteria must be one of the causes of excessive and abnormal cell proliferation in crown gall.” It was then thought that *A. tumefaciens* was invasive and penetrated into plant host tissues.

### *A. tumefaciens* produces phytohormones that caused tumor growth

**Figure 5:**
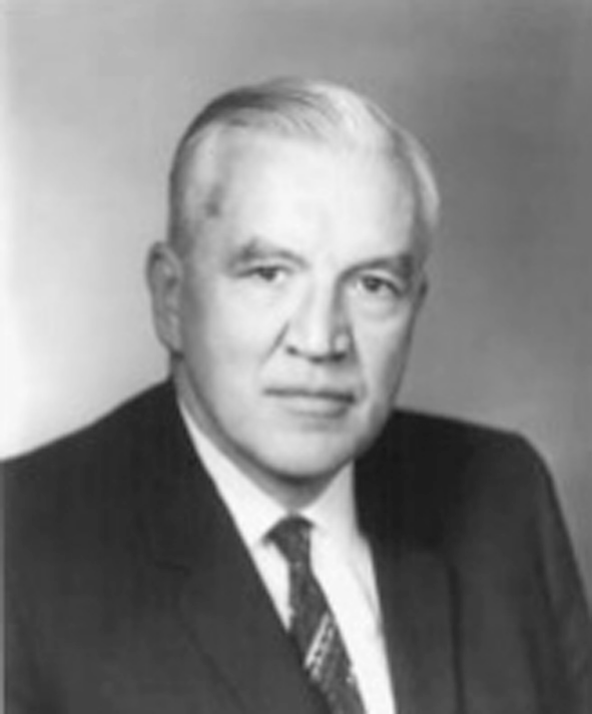
**F. W. Went, the discoverer of auxin**.

By the late 1920s, a plant growth substance named auxin (Went, 1926; Figure 5) was believed to play a key role in tumor growth as it was stated that “The auxin swellings bear close resemblance to the phenomena observed in some of the galls and other pathological outgrowths and there is good evidence that auxin plays an important part in such growths” (Went and Thimann, 1937). The auxin indole-3-acetic acid was found in human urine and produced by various fungi and bacteria (reviewed in Went and Thimann, 1937). Its production in plants was first confirmed in oat coleoptiles (*Avena sativa*) (Went, 1928). Subsequently, several investigators noted similarities between the reaction of plant tissues treated with indole-3-acetic acid produced by *A. tumefaciens* from tryptophan and the reaction of similar plant material inoculated with the pathogen itself (Brown and Gardner, 1936; Kraus et al., 1936; Link et al., 1937). Plant host tissue swellings and gall-like outgrowths were obtained by applying extracts from cultures of *A. tumefaciens* (then called *P. tumefaciens*) (Brown and Gardner, 1936). Using an attenuated culture of *A. tumefaciens* (then called *P. tumefaciens*), Braun and Laskaris (1942) found that the avirulent strain was capable of inducing tumors closely resembling crown gall on tomato plants when the bacteria were supplemented with either α-naphthalene acetic acid, γ-indole butyric acid, or β–indole acetic acid. These workers stated that “The discovery that synthetic growth substances were able to stimulate the development of tumors by the attenuated culture strengthened our previous belief regarding the probable role of the host growth hormones in the development of these neoplastic growths.” This was somewhat contrary to the work of Locke et al. (1938) who tested an attenuated strain on decapitated tomato and *Bryophyllum* plants treated with 30 mg indole-3-acetic acid per gram of lanolin paste at the cut site and found that “… there was a slight stimulation in plants treated with the acid over untreated plants.” Interestingly, these workers noticed “… the galls from virulent cultures were without chlorophyll while those from attenuated cultures were green.” Based on the positive effects of phytohormones on the avirulent strain leading to tumor growth and the continued tumor growth of implanted tissue fragments from tumors initiated by the attenuated *A. tumefaciens* strain stimulated with phytohormone, Braun and Laskaris (1942) proposed that there appear to be at least two distinct phases involved in tumor formation. The first phase involves stimulation of normal cells. The second phase requires continued stimulation resulting in cellular multiplication by a growth substance, resulting in tumor formation (Braun, 1952). This premise appears to be the combination of the above two concepts, i.e., the need for a chemical irritant and the presence of phytohormones.

### Permanent autonomous growth of crown gall tissue *in vitro*: first clues that a genetic change has occurred

**Figure 6:**
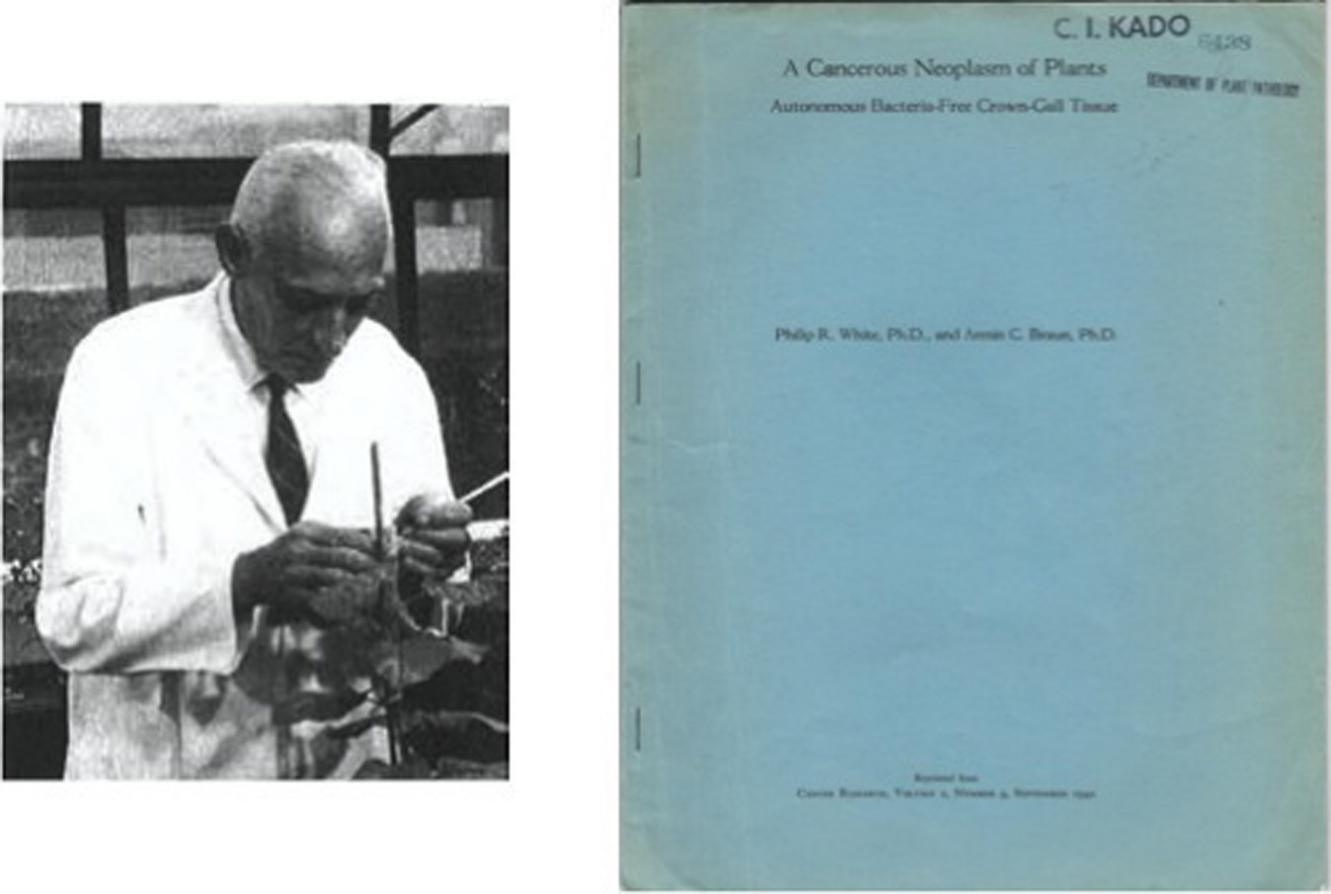
**Photo of Armin C. Braun in his greenhouse laboratory**. Classic paper on auxin autonomy of crown gall tissue culture by White and Braun (1942).

**Figure 7:**
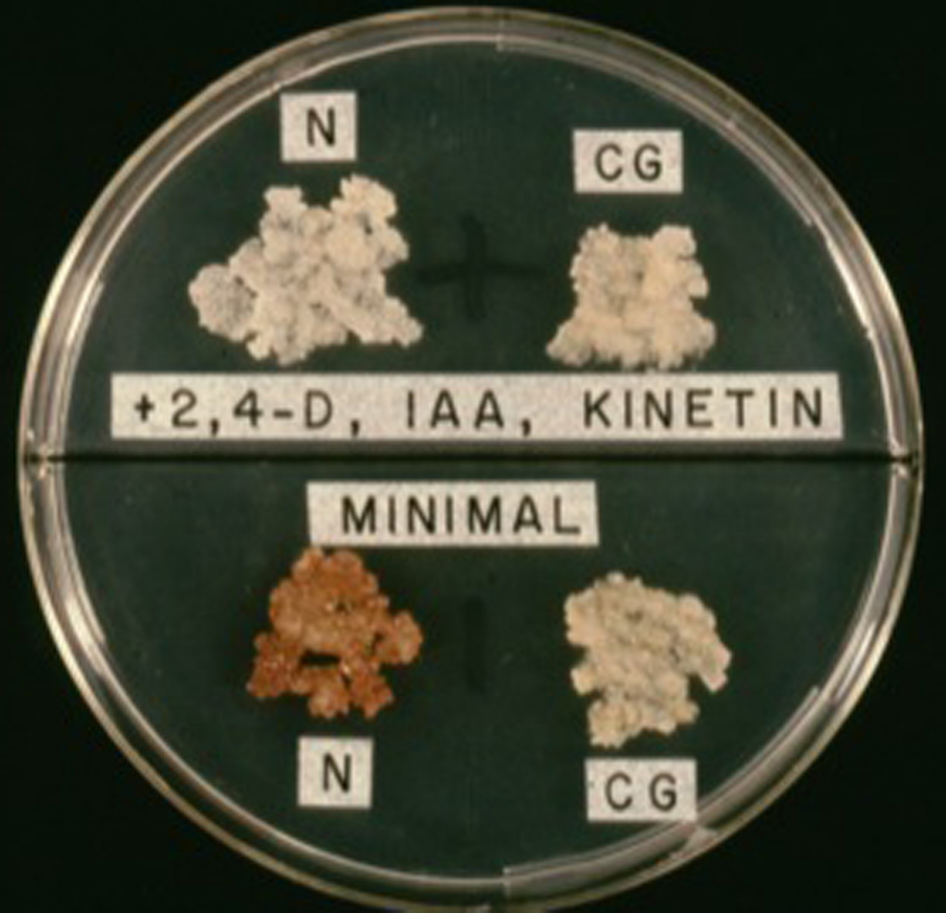
**Auxin autotrophy of crown gall tissues on hormone-free medium (lower half of bisectored petri plate)**. N, normal cells; CG, crown gall cells; 2,4-D, 2,4-phenoxyacetic acid; IAA, indole-3-acetic acid.

One of the most significant discoveries that have led to our current understanding of the mechanism by which *A. tumefaciens* causes crown gall was the work of White and White and Braun (1942; Figure 6) and Braun and White (1943). These workers showed that crown gall tumors derived from secondary tumors were bacteria-free, as determined by cultural and serological methods. This finding brought forth the idea that there was indeed some form of genetic transformation of the host plant cell that was infected by *A. tumefaciens*. Significantly, the isolated crown gall tumor tissues grew well in the absence of phytohormones (Figure 7). Hence, they were autonomous with respect to the need of phytohormones (auxin-autotrophic) that normal plant tissues in culture required for growth.

Further indirect evidence that a genetic transformation has taken place in crown gall is derived from the presence of rare guanidine derivatives such as octopine and nopaline in crown gall tissues. The *A. tumefaciens* strain B6 that metabolize octopine was found also to induce tumors that contained octopine (Menagé and Morel, 1964; Goldmann-Ménagé, 1971; Morel, 1972). Likewise, *A. tumefaciens* strains that metabolize nopaline induced tumors that produced nopaline (Goldmann et al., 1969). These guanidine compounds appear to be determined exclusively by the type of *A. tumefaciens* strain used to induce crown gall and are not dependent on the plant species (Petit et al., 1970; Bomhoff, 1974). However, Wendt-Gallitelli and Dobrigkeit (1973) found octopine in habituated tobacco cells, and in the root tips of young pea and bean seedlings. These workers concluded that because of the presence of this guanidine derivative in non-transformed plant material, octopine is not exclusive to crown gall tumors. Earlier work showed that lysopine is present only in crown gall tumor tissues (Lioret, 1956). However, Seitz and Hochster (1964) found it to be produced in small amounts in normal tobacco and tomato plants. Also, Johnson et al. (1974) detected octopine in normal tobacco, sunflower, pinto bean and tobacco callus tissues. Although trace amounts of unusual guanidine compounds had been detected in the above plants, opines such as octopine and nopaline exclusively occur in crown gall tissues.

Given these suggestions that *Agrobacterium* genetically transforms plants, the idea that DNA might be transferred from *A. tumefaciens* into the plant cell became a popular notion. Hence, a number of workers proposed that crown gall induction involves the transfer of bacterial DNA into plant cells (Milo and Srivastava, 1969; Quétier et al., 1969; Schilperoort, 1969; Srivastava, 1970; Srivastava and Chadha, 1970; Chadha and Srivastava, 1971; Stroun et al., 1971; Yajko and Hegeman, 1971; Heyn and Schilperoort, 1973). However, this enthusiasm was dampened when other workers failed to induce crown gall tumors by introducing purified DNA from *A. tumefaciens* into plants (Braun and Wood, 1966; Bieber and Sarfert, 1968; Stroun et al., 1971; Yajko and Hegeman, 1971).

**Figure 8:**
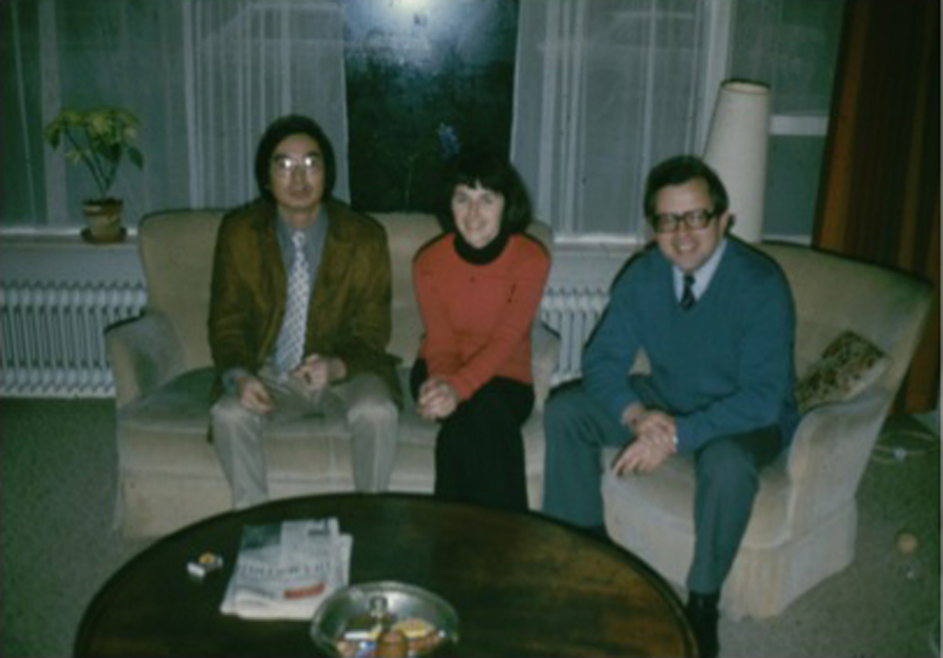
**Rob Schilperoort (right), his wife (middle) with Clarence Kado (left)**.

Although bacteriophages had been found in axenically grown crown gall tissues (Tourneur and Morel, 1971), an interesting report claimed that DNA of an *A. tumefaciens* bacteriophage called PS8 was present as a plasmid in crown gall tumor cells (Schilperoort, 1969; Schilperoort et al., 1973; Figure 8). Also, Schilperoort (1971) found strong complementarity of *A. tumefaciens* cRNA to crown gall tissue DNA. This work could not be verified either by Eden et al. (1974), or by Farrand et al. (1975) who used DNA/RNA filter hybridization and by Chilton et al. (1974) who used renaturation kinetics in an attempt to detect bacterial and phage DNA in crown gall tumors. They stated that they “… found no evidence for bacterial or phage DNA in the tumors examined.” Drlica and Kado (1974) used DNA:DNA filter hybridization and solution enrichment techniques and found that no more than 0.02% of the crown gall tumor genome could contain *A. tumefaciens* DNA. This work left open the possibility that some traces of *A. tumefaciens* DNA might be incorporated into the plant host cell genome.

Kado and Lurquin (1976) established that exogenously added naked *A. tumefaciens* DNA to cultured tobacco cells is not stably maintained in the plant cells and nuclei. Braun and Wood (1966) found that the addition of deoxyribonuclease (DNase) at concentrations up to 5 mg/ml was completely ineffective in inhibiting tumor inception or development when the enzyme solution was applied 1–2 h prior to the time that the plants were inoculated with *A. tumefaciens* or when the bacterium and DNase were added to the wound site together. Interestingly, Braun and Wood (1966) reported that ribonuclease A (RNase) inhibited tumor formation when high concentrations (2–4 mg/ml) of the enzyme solution were applied 1–2 h prior to the time that the wound site was inoculated with *A. tumefaciens*. RNase neither affected bacterial growth, nor the virulence of the bacterium, nor the wound-healing process. These early studies suggested that the bacterial DNA must gain entry into plant cells in a protective fashion. Hence, it remained possible that bacterial-specific DNA might be passed to plant cells via some form of intimate bacteria-plant cell interaction. The above studies on subjecting plant cells to naked *A. tumefaciens* DNA indicate that the release of naked DNA by *A. tumefaciens* and its uptake by plants are not the process of plant cell transformation.

So, how is bacterial DNA transferred to plant cells? If transferred, is the DNA encapsulated or protected in some way in order to survive the transfer process? These were some of the important questions asked during that period when not much was understood about the plant-microbe interaction. Researchers began investigating how *A. tumefaciens* perceives its plant host, how it might attach to the host tissues, how it would transfer DNA and how the transferred DNA is processed in the host cells. There apparently is the absence of specific receptors on plant protoplasts onto which *A. tumefaciens* might bind and insert its DNA (Schilde-Rentschler, 1973), so binding of bacterial cells, if at all, must be at sites other than protoplastic membranes. Because wounding was required to initiate tumor formation, the plant cell wall was thought to be a barrier against effective transformation by bacteria cells. Hence, Virts and Gelvin (1985) infected *Petunia* protoplasts with *A. tumefaciens* and found bacterial DNA transferred within 2–6 h into the plant cell but most of the DNA was rapidly degraded. Earlier, Schilperoort (1969) observed attachment of bacteria to intact plant cells and later work by Krens et al. (1985) found that tobacco leaf protoplasts regenerating primary cell wall could be transformed by co-cultivation with intact *A. tumefaciens*. Apparently bacterial cellulose fibrils appear to play a role in attachment (Matthysee, 1986).

The important question arose whether or not foreign circular DNA would survive in plant cells. That question was answered by the experiments of Lurquin and Kado (1977). These workers showed that plasmids such as pBR313, a covalently closed DNA, could be taken up by plant protoplasts and remain intact in the nucleus for extended periods of time. Kerr (1969, 1971) observed that oncogenicity could be transferred from one strain of *A. tumefaciens* to another by inoculating both strains together or in succession onto the same plant. Hamilton and Chopan (1975) established that non-pathogenic strains of *A. radiobacter* or *A. tumefaciens* were converted to pathogens by surface inoculation of developing crown galls that harbored the transforming and virulent *A. tumefaciens*. The co-inoculation technique described by Kerr (1969, 1971) was called the “Kerr-cross.” Although there was no definitive idea on how virulence was transferred, Roberts and Kerr (1974) elegantly stated that “… it would seem that the only other likely method of DNA transfer is through conjugation.” It was well established that Hfr strains of *Escherichia coli* could transfer genetic information to *Salmonella typhimurium* (Baron et al., 1959; Miyake and Demerec, 1959). Likewise, Mitsuhashi (1977) found plasmids, called R factors, conferring antibiotic resistance that could transfer between different bacterial species via a conjugative process. Hence, the question was raised as to whether or not *A. tumefaciens* contained a conjugative plasmid.

This question was indirectly answered by an observation made by Hamilton and Fall (1971). These workers noticed *A. tumefaciens* strains C58 and Ach5 lost their virulence when sub-cultured for 5 days at 36°C. Temperatures above 31.5°C or exposure to ethidium bromide resulted in either the loss of a large plasmid or deletion of a portion of the large plasmid leading to the loss of virulence in *A. tumefaciens* (Lin and Kado, 1977). Interestingly, Braun and Mandle (1948) earlier found that 32°C was the temperature that completely stopped the transformation of normal cells to crown gall tumor cells following inoculation by *Agrobacterium*.

**Figure 9:**
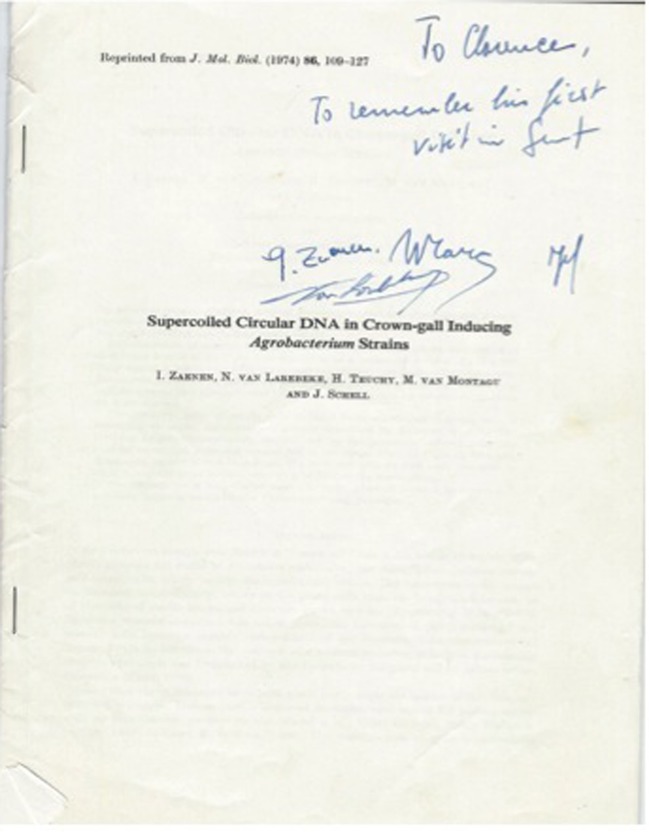
**Classic paper first reporting the presence of an *A. tumefaciens* plasmid associated with tumorigenicity by Jeff Schell's laboratory**.

**Figure 10:**
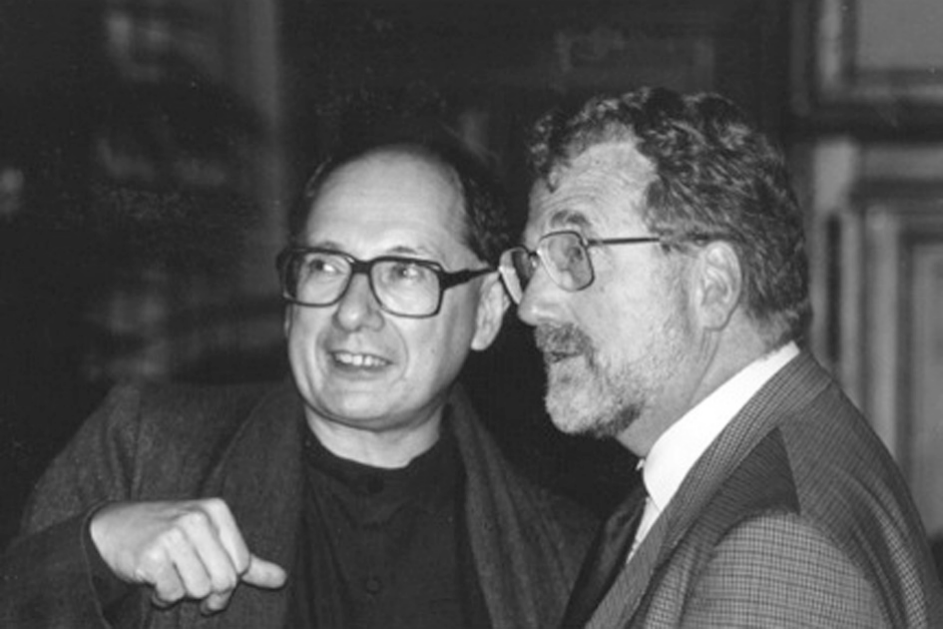
**Marc van Montagu and Jeff Schell**.

The importance of bacterial plasmids was confirmed by the detection and isolation of large extrachromosomal elements in virulent strains of *A. tumefaciens* but not in *A. radiobacter* strains (Zaenen et al., 1974; Figure 9). We had earlier explored the possibility of the existence of a plasmid in *A. tumefaciens* but failed to find any owing to the use of a plasmid isolation technique developed for *E. coli* rather than for *A. tumefaciens* (Kado et al., 1972). Interestingly, other workers showed that both large and small plasmids exist in both virulent *A. tumefaciens* and *A. radiobacter* strains (Merlo and Nester, 1977; Sheikholeslam et al., 1978). Zaenen et al. (1974) examined eight different avirulent strains and found none of them harbored large plasmids. The curious absence of large plasmids in those avirulent strains of *A. tumefaciens* or *A. radiobacter* examined by Zaenen et al. (1974; Figure 10) was believed to be a lucky choice of strains according to Jeff Schell (pers. commun. 1978). The conversion of virulent *A. tumefaciens* to stable avirulent strains by sub-culturing at elevated temperatures (32–37°) was shown to be due to the concomitant loss of a large plasmid (Watson et al., 1975). Moreover, by using the “Kerr cross” technique, an avirulent strain of *A. tumefaciens* was shown to acquire tumor-inducing ability by acquiring a 58 μm plasmid (Van Larebeke et al., 1975).

**Figure 11:**
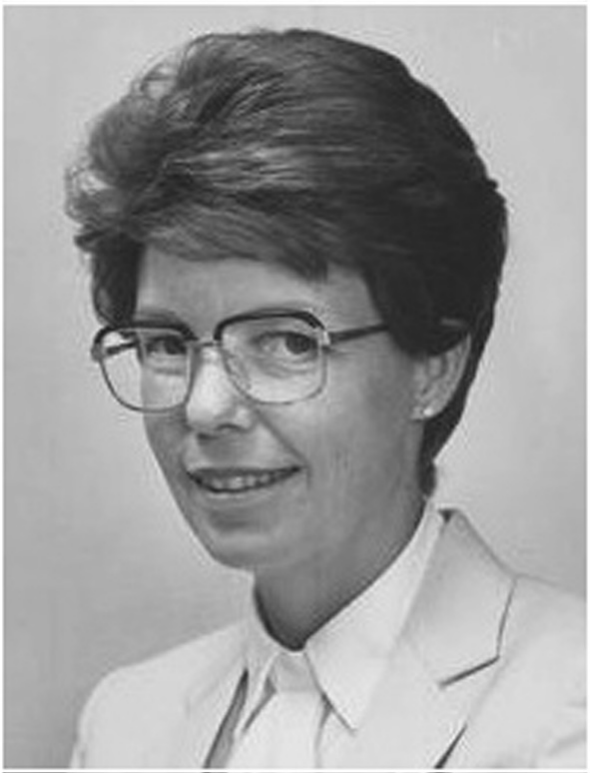
**Mary-Dell Chilton**.

The presence of a plasmid that conferred virulence upon *A. tumefaciens* led to investigations seeking plasmid DNA in crown gall cells. Indeed, Chilton et al. (1977; Figure 11) detected trace amounts of a part of the plasmid in crown gall cells. The amount of foreign DNA represented 0.0011% of total DNA content of the tumor cell. This was a very significant discovery since no other bacterial pathogen has been shown to transfer DNA to plant cells.

## An extrachromosomal element confers virulence on *A. tumefaciens*

The establishment that a large *A. tumefaciens* plasmid called the Ti plasmid (for tumor-inducing) confers virulence initiated a large number of studies on identifying plasmid genes that were transferred to the host plant cell as well as identifying the intrinsic properties of the large plasmid harbored in virulent strains of *A. tumefaciens* (Gelvin, 2000). As history of these studies show (Nester et al., 2005), emphasis shifted toward developing an understanding of the mechanism of horizontal gene transfer (HGT) by *A. tumefaciens* since this organism represent the first valid case of the inter-domain gene transfer (Bacteria to Eukarya) (Kado, 2009).

Although a number of ancillary studies on the Ti plasmid were started, the initial main efforts were on mapping the location of genes required for conferring the tumor-inducing properties on *A. tumefaciens*. The Ti plasmid of octopine strain B6-806 was physically mapped using restriction endonucleases (Chilton et al., 1978b; Koekman et al., 1979). The Ti plasmid of the nopaline strain C58 was similarly mapped by restriction endonuclease analysis (Depicker et al., 1980). Both deletion-mutational and transposon-insertional mapping were used to locate genes encoding known octopine and nopaline Ti plasmid phenotypes (Holsters et al., 1980; Degreve et al., 1981; Garfinkel et al., 1981). Southern blot analysis and heteroduplex mapping were used to identify homologous “common region” and non-homologous sequences between the octopine plasmid pTiAch5 and the nopaline plasmid pTiC58 (Engler et al., 1981). Altogether, two EcoRI fragments present in the nopaline Ti plasmid pTiC58 and homologous to a segment of the octopine plasmids pTiB6S3 and pTiAch5 identified the region that confers oncogenicity on *A. tumefaciens* (Chilton et al., 1978a,b; Depicker et al., 1978; Schell et al., 1979). DNA reassociation kinetic analyses were used to probe four tumor lines induced by three *A. tumefaciens* strains (Merlo et al., 1980). This study revealed that a specific sector of Ti plasmid DNA, called the T-DNA (for transferred DNA) coincides with the same region of the physical map of the plasmids. The length of the T-DNA was found to vary in different tumor lines and is flanked on each end by 25 base-pair repeated sequences (Yadav et al., 1982; Wang et al., 1984). The T-DNA borders are similar to the sequences of broad and narrow host-range plasmids that are recognized by their respective nicking enzymes (Kado, 1998). The processing of the T-DNA, initiated by nicking or cleavage at the T-DNA borders, has nicely reviewed by Zambryski (1992) and Gelvin and Filichkin (1994). The right border sequence is essential for and determines the direction of DNA transfer from *Agrobacterium* to the plant genome (Wang et al., 1984). The transfer of the T-DNA, as a single-stranded molecule (Stachel et al., 1986), by a conjugal mechanism is discussed below and recently reviewed by Gelvin (2012). T-DNA is localized to the nucleus of host plant cells and covalently linked to the nuclear DNA (Chilton et al., 1980; Willmitzer et al., 1980).

The next obvious objective regarding the T-DNA was to identify its encoded functions in crown gall tumor cells. At least six discrete T-DNA-encoded mRNAs of sizes 0.73–1.75 kb were detected in octopine-producing tumor lines (Gelvin et al., 1982; Willmitzer et al., 1982) and sizes 0.67–2.7 kb were detected in nopaline tumor lines (Willmitzer et al., 1982). Polyadenylated mRNA transcribed from the T-DNA revealed a transcript 2 (designated earlier as *tms-2* revised as *iaaH*) that is directly responsible for the production of indole-3-acetic acid from indole-3-acetamide, whose formation is catalyzed by indoleacetamide hydrolase from tryptophan (Inze et al., 1984; Schröder et al., 1984). Transcript 1 (designated as *iaaM*) encodes tryptophan 2-monooxygenase (Van Onckelen et al., 1986). Several workers had reported that cytokinin biosynthesis was associated in some way with the T-DNA (Akiyoshi et al., 1983; Barry et al., 1984; Buchmann et al., 1985). The biochemical pathways for auxin and cytokinin have been reviewed (Morris, 1986). Although the T-DNA is weakly transcribed in *A. tumefaciens* (Gelvin et al., 1981), the *ipt* gene located in the T-DNA that encodes isopentenyltransferase activity is not fully expressed in *A. tumefaciens* (Heinemeyer et al., 1987). It was later shown that the *Ipt* gene was repressed by a eukaryotic-like zinc-finger protein called Ros encoded by the chromosomal *ros* gene of *A. tumefaciens* (Chou et al., 1998) and derepressed by a single amino acid substitution of Ros (Archdeacon et al., 2006). Nuclear magnetic resonance spectroscopic studies of Ros revealed a novel DNA recognition mechanism of eukaryotic promoters (Malgieri et al., 2007).

Besides phytohormone genes in the T-DNA, opine synthase genes are also located within the T-DNA. The nopaline synthase gene (*nos)* is located near the right border of the T-DNA (Depicker et al., 1982; Joos et al., 1983). The octopine synthase encoded by the *ocs* gene located in the T-DNA of octopine Ti plasmids was characterized biochemically (Schröder et al., 1981). A gene that encodes agrocinopine synthase was also located in the T-DNA of nopaline Ti plasmids, and a gene that encodes agropine synthase was identified in octopine Ti plasmids (Joos et al., 1983; Paulus and Otten, 1993). These opine synthase genes are integrated into the plant host genome. The opines produced are generally condensation products between basic amino acids and organic acids such as between arginine and pyruvate (octopine). Opines can serve as carbon and sometimes nitrogen compounds utilized by *A. tumefaciens* for nutritional and Ti-plasmid conjugational activities (reviewed in Dessaux et al., 1991; Farrand, 1993). The specificity of opine utilization by *A. tumefaciens* is not entirely tight since fluorescent *Pseudomonas* spp. associated with crown gall tumors in the field appear to catabolize opines (Moore et al., 1997).

After *Agrobacterium*-mediated transformation, these opine synthase genes are transferred to plant hosts by *A. tumefaciens*. This prompted in-depth studies on the T-DNA processing and transfer system. This historical review will not cover this aspect of the biology of crown gall. The processing and transfer of the single-stranded T-DNA covalently linked to VirD2 and then bound with VirE2 (T-DNA complex) was nicely reviewed in Zambryski et al. (1989), Zambryski (1992), Hansen and Chilton (1999), Gelvin (2003), Citovsky et al. (2007).

Gaining detailed insights on the functions expressed by T-DNA genes directed efforts to another sector of the plasmid (designated as the *vir* region) that was required for virulence. Through initial genetic analyses (Tn5- and Tn3-lacZ induced mutagenesis) (Garfinkel and Nester, 1980; Stachel and Nester, 1986) and DNA sequencing of the *vir* region, it was initially determined that there were six operons, designated as VirA, B, G, C, D, and E, arranged in that sequential order, as a *vir* regulon (Rogowsky et al., 1990; Schrammeijer et al., 2000; Hattori et al., 2001). Each operon in the vir regulon contains the box sequence (TNCAATTGAAAPy) for both octopine and nopaline Ti plasmids (Steck et al., 1988). A *vir* gene designated *virF* was found in octopine strain A6 that confers host specificity and restricts T-DNA transfer to maize (Jarchow et al., 1991). Every *vir* operon plays an important role either in facilitating bacterial recognition of its host plant through distal and proximal interactions (Rogowsky et al., 1987; Winans, 1992), or generating a T-DNA delivery and processing system (Hooykaas and Beijersbergen, 1994). Expression of the *vir* operon is initiated by sensory detection of external chemical inducers such as acetosyringone (Stachel et al., 1985) and sinapinic acid (Rogowsky et al., 1987). Inter-communications between *Agrobacterium* and its plant host by means of chemical signals, such as precursors of lignin biosynthesis (phenols), sugars and acidic conditions in plants, leading to expression of Ti plasmid virulence genes have been extensively reviewed by Winans (1992) and Gelvin (2006). Much of the early studies focused on the encoded functional roles of *vir* genes within each operon, and those involved in T-DNA processing and its transfer to the host plant cell were key players (reviewed in Gelvin, 2012).

The early prediction by Roberts and Kerr (1974) that *A. tumefaciens* must use a conjugative process to deliver oncogenes appears to be insightfully correct. Furthermore, the prediction was made that the transfer of T-DNA from *A. tumefaciens* to plants is a conjugative system requiring a “sex” pilus (Kado, 1994). Concerted efforts by several research groups carefully analyzed the functional role of the *virB* operon (Shirasu and Kado, 1993a; Jones et al., 1996; reviewed in Zupan et al., 1998). These analyses of the VirB operon revealed striking similarities in both gene organization and sequences to genes involve in conjugative transfer of broad-host-range plasmids (Shirasu and Kado, 1993b). The *virB2* genes sequence shows similarities to *traA* of the enteric plasmid F and to *trbC* of the PilW operons of plasmid R388. The striking similarities between VirB2 and TraA in their amino acid sequences, their protein processing into a 7.2-KDa subunit, and their location in the bacterial cell brought forth the hypothesis that *virB2* encodes a VirB2 pilin subunit used in the transfer of the T-DNA (Shirasu and Kado, 1993a). Consequently, efforts were made to search for pili made by *A. tumefaciens*. A pilus of 3 nm diameter was reported by Fullner et al. (1996). Careful analyses revealed that both virulent and avirulent *A. tumefaciens* produce a common pilus of 3 nm diameter, but only the virulent induced strain, lacking the interfering flagella (Chesnokova et al., 1997), produced a long pilus of 10 nm diameter with a 2-nm lumen (Lai and Kado, 1998, 2000, 2002). This pilus was named the “T-pilus” (Lai and Kado, 1998, 2000).

The products of the *virB* operon are required for oncogenesis and associate with the inner and/or outer membrane of *A. tumefaciens*. The membrane association of these products was thought possibly to form some type of transport system that was distinguished as a member of the type IV secretion system (Christie, 1997; O'Callaghan et al., 1999; Christie et al., 2005). This secretion machinery is involved in the transport of the T-DNA-VirD2 complex (reviewed in Zupan et al., 1998). Comparisons between various known type IV secretion systems have revealed a high degree of conservation in their structural features (Zechner et al., 2012). Besides the type IV secretion machinery involving *virB2* genes, Ti plasmid and genomic sequence analyses have revealed two additional type IV secretion systems in *A. tumefaciens*, one of which is required for conjugative transfer of the cryptic plasmid pAtC58 (Chen et al., 2002) and the other, designated as the Trb locus is required for conjugal transfer of the Ti plasmid (Von Bodman et al., 1989; Li et al., 1999). Moreover, recent work on *Agrobacterium* secretion systems has demonstrated the presence of a type VI secretion machinery having little effect on virulence. However, it may play an ancillary role in facilitating virulence (Wu et al., 2008), as do type VI secretion systems in other pathogens equipped with this secretory system to counter-act intruding competing bacteria (Basler et al., 2013). In addition, the type VI machinery is reported to translocate a phage tail spike-like protein into target cells, cross-link with actin and serve as a tool to puncture membranes of the host cell (Pukatzki et al., 2007).

**Figure 12:**
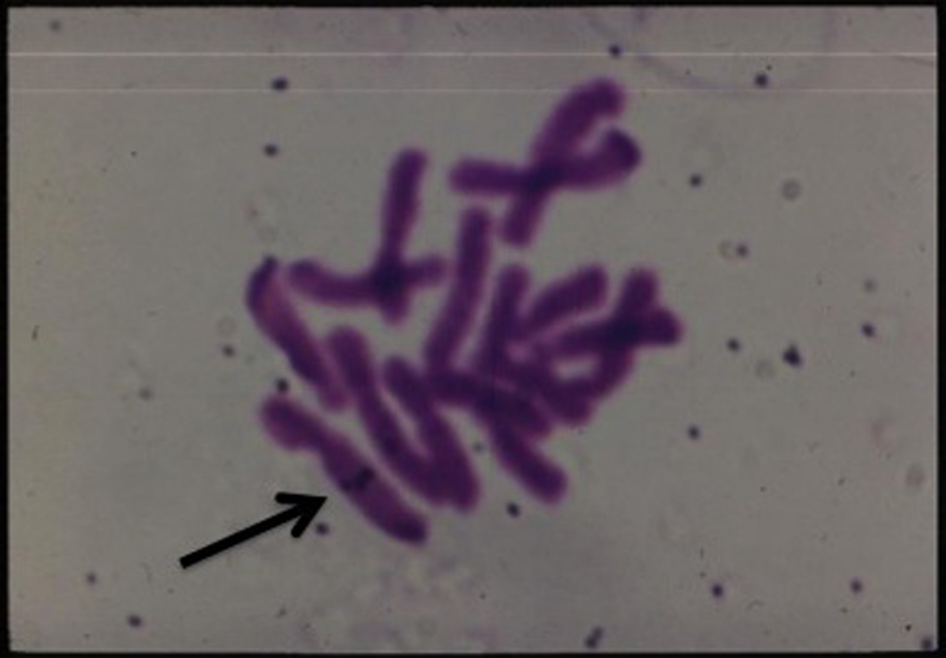
***In situ* hybridization of labeled T-DNA integrated in the chromosome of *Happlopappus gracilis* (Quayle and Kado)**.

Lastly, but not the least is the insightful work accomplished on determining the fate of the transferred T-DNA complex culminating in its integration in the nuclear chromosomal DNA of the host (reviewed by Tzfira et al., 2004; Lacroix and Citovsky, 2009; Figure 12).

Accessory chromosomal genes assist in facilitating virulence and regulating both genes of the *vir* regulon and the T-DNA of *A. tumefaciens* (reviewed in Charles and Nester, 1994). DNA sequencing of the bacterial circular and linear chromosomes helped locate these genes of potential significance in tumorigenesis (Wood et al., 2001; Goodner et al., 2011; Slater et al., 2013). Additional comparative sequence analyses between *A. tumefaciens* strains of limited host ranges may still reveal novel genes conferring host and ecological (environmental) specificity.

## Conclusions and perspectives

The historical event of finding and isolating a tumor-inducing bacterium from grapevine galls (Cavara, 1897a,b) initiated a wonderful, long journey of scientific research that has led to our understanding and appreciation on how *A. tumefaciens* evolved to be equipped with some very sophisticated means of surviving in a hostile soil environment and on plants (Palumbo et al., 1998). This organism escaped numerous microbial competitors such as *Pseudomonas aeruginosa*, *P. fluorescens*, *Streptomyces* spp. (Hibbing et al., 2010) by swimming away from the competition (An et al., 2006) and establishing its own niche in plants in the form of overgrowths (tumors) and essentially genetically engineering the plant host to provide highly specialized organic compounds (opines) that could be specifically utilized by the tumor-inducer. An evolutionarily built-in DNA escape mechanism of purely selfish nature (Orgel and Crick, 1980) as exemplified by the conjugal chromosomal, Ti plasmid and T-DNA transfer to other microbes and to plants (Fründt et al., 1998) insured its survival. *A. tumefaciens* represents the first *living* representative of HGT, i.e., transfer between the domains Bacteria and Eukarya (Kado, 1998, 2009).

All of the pioneering research groups that contributed to these biological understanding of *A. tumefaciens* and crown gall should be applauded.

Furthermore, it is well established that applications of the HGT system between bacteria and plants (Caplan et al., 1983; Fraley et al., 1985) has led to major commercial applications that yielded many genetically engineered domesticated crop (food and fiber) plants as well as serving as a tool for investigating plant immunity responses, plant disease control through transfer of iRNA, etc. *A. tumefaciens* represents and continues to be a valuable resource for biotechnology and humanity.

### Conflict of interest statement

The author declares that the research was conducted in the absence of any commercial or financial relationships that could be construed as a potential conflict of interest.
